# RACIAL DISPARITIES IN HIP FRACTURE CARE

**DOI:** 10.1093/geroni/igz038.1750

**Published:** 2019-11-08

**Authors:** Iman Ali, Jessica Jang, Sanjana Vattigunta, Ankit Bansal, Uma Srikumaran

**Affiliations:** 1 Johns Hopkins University, Baltimore, Maryland, United States; 2 Johns Hopkins, Baltimore, Maryland, United States

## Abstract

Hip fractures are associated with significant morbidity and mortality. Delaying surgery for more than 24 hours after presentation results in more complications, higher 30-day mortality rate, and longer stays in the hospital. As such, high-quality care should be provided consistently to an increasingly diverse patient population. We determined if race characteristics influence the quality of care provided to patients with hip fractures. We conducted a retrospective analysis on patients at our institution between January 2015 and December 2017. Patients were categorized as white, Black, Asian, and other. The primary outcome variable was the time between presentation to surgery. Other outcomes included length of hospital stay and narcotic pain medication consumption in the first 24 hours postoperatively. Adjusted analysis was performed, controlling for sex, age, body mass index (BMI), American Society of Anesthesiologists’ (ASA) classification of health, and Charlson Comorbidity Index (CCI). There were 1544 hip fracture patients included in the study. The majority of patients were white (84.1%) followed by Black (7.6%), Asian (4.5%), and other (3.7%). Most patients were female (69.6%). After adjusting for patient characteristics, Black patients experienced a significantly greater delay to surgery after presentation than white patients (42.1 vs. 34.9 hours). In addition, Black patients experienced significantly longer length of hospital stays compared to their white counterparts (6.9 vs. 5.8 days). Racial disparities in the quality of care provided to hip fracture patients persist even after adjusting for patient characteristics. Addressing these disparities can possibly enhance outcomes for minority patients.

